# Awareness of physical activity in healthy middle-aged adults: a cross-sectional study of associations with sociodemographic, biological, behavioural, and psychological factors

**DOI:** 10.1186/1471-2458-14-421

**Published:** 2014-05-02

**Authors:** Job G Godino, Clare Watkinson, Kirsten Corder, Stephen Sutton, Simon J Griffin, Esther MF van Sluijs

**Affiliations:** 1 MRC Epidemiology Unit, Institute of Metabolic Science, University of Cambridge, Addenbrooke's Hospital, Box 285, Hills Road, Cambridge CB2 0QQ, United Kingdom; 2 UKCRC Centre for Diet and Activity Research (CEDAR), Institute of Public Health, University of Cambridge, Box 296, Forvie Site, Robinson Way, Cambridge CB2 0SR, United Kingdom; 3 Behavioural Science Group, Institute of Public Health, University of Cambridge, Forvie Site, Robinson Way, Cambridge CB2 0SR, United Kingdom

**Keywords:** Physical activity, Objective measurement, Awareness, Misperception, Barriers, Correlates, Behaviour change, Personalised feedback

## Abstract

**Background:**

Interventions to promote physical activity have had limited success. One reason may be that inactive adults are unaware that their level of physical activity is inadequate and do not perceive a need to change their behaviour. We aimed to assess awareness of physical activity, defined as the agreement between self-rated and objective physical activity, and to investigate associations with sociodemographic, biological, behavioural, and psychological factors.

**Methods:**

We conducted an exploratory, cross-sectional analysis of awareness of physical activity using baseline data collected from 453 participants of the Feedback, Awareness and Behaviour study (Cambridgeshire, UK). Self-rated physical activity was measured dichotomously by asking participants if they believed they were achieving the recommended level of physical activity. Responses were compared to objective physical activity, measured using a combined accelerometer and heart rate monitor (Actiheart®). Four awareness groups were created: overestimators, realistic inactives, underestimators, and realistic actives. Logistic regression was used to assess associations between awareness group and potential correlates.

**Results:**

The mean (standard deviation) age of participants was 47.0 (6.9) years, 44.4% were male, and 65.1% were overweight (body mass index ≥ 25). Of the 258 (57.0%) who were objectively classified as inactive, 130 (50.4%) misperceived their physical activity by incorrectly stating that they were meeting the guidelines (overestimators). In a multivariable logistic regression model adjusted for age and sex, those with a lower body mass index (Odds Ratio (OR) = 0.95, 95% Confidence Interval (CI) = 0.90 to 1.00), higher physical activity energy expenditure (OR = 1.03, 95% CI = 1.00 to 1.06) and self-reported physical activity (OR = 1.13, 95% CI = 1.07 to 1.19), and lower intention to increase physical activity (OR = 0.69, 95% CI = 0.48 to 0.99) and response efficacy (OR = 0.53, 95% CI = 0.31 to 0.91) were more likely to overestimate their physical activity.

**Conclusions:**

Overestimators have more favourable health characteristics than those who are realistic about their inactivity, and their psychological characteristics suggest that they are less likely to change their behaviour. Personalised feedback about physical activity may be an important first step to behaviour change.

## Background

Regular engagement in physical activity offers many well-established health benefits, including reduced risk of obesity, type 2 diabetes, cardiovascular disease, and some cancers [1]. According to previous national and international guidelines, adults should accumulate at least 30 minutes of moderate to vigorous physical activity on 5 or more days of the week in order to derive such benefits (current guidelines encourage adults to engage in moderate-intensity aerobic physical activity for at least 150 minutes per week) [2-4]. Public health campaigns have been implemented to educate the general population about the wide-ranging benefits of physical activity and to increase awareness of the guidelines [5,6]. Despite these efforts, accelerometry data from the UK and the US indicate that fewer than 5% and 10% of the adult population are achieving the recommended level of physical activity, respectively [7,8]. The proportion of adults who meet the guidelines is unlikely to increase without the development of more effective physical activity promotion efforts. Multiple systematic reviews have concluded that existing interventions implemented in primary care and community settings are limited in that they produce only small, short-term changes in behaviour [9-12]. Reasons for this general lack of effectiveness remain unclear [13].

One hypothesis is that inactive adults do not perceive a need to change their level of physical activity because they are unaware that their current behaviour is inadequate [14-17]. Evaluating the adequacy of one’s physical activity is difficult because it not only requires an accurate summarisation of the frequency, duration, and intensity of activity into a single metric, but also knowledge of what constitutes a healthy level. In contrast to dichotomous behaviours, such as smoking or condom use, physical activity includes numerous planned, habitual, and incidental (e.g., walking, stair climbing, or standing) activities throughout one’s day [17]. This makes the distinction between healthy and unhealthy behaviour less clear, and such a distinction may be complicated by inconsistent or changing guidelines [18].

Awareness of physical activity has previously been defined as the agreement between self-rated and actual levels of physical activity. Self-rated physical activity is assessed by asking individuals to provide a single evaluation of the quantity of physical activity they engage in (e.g., “active” or “inactive”). Actual levels of physical activity are assessed using either a self-reported measure (e.g., a physical activity questionnaire) or an objective measure (e.g., an accelerometer), both of which result in a quantified level of physical activity. It is important to highlight that the discrepancy assessed in measures of awareness of physical activity represents the accuracy of an individual’s belief about whether or not their level of physical activity is adequate, and is distinct from determining error or validity when comparing self-reported and objective measures.

Previous studies of the awareness of physical activity show that as many as 61% of adults who are not achieving the recommended level of physical activity overestimate their activity [14-17]. Although their inactivity places them at increased risk of a variety of chronic diseases and disorders [1], overestimators tend to have more favourable health characteristics compared to those who are realistic about their inactivity [15-17]. Notably, they are also less likely to express an intention to change their behaviour [14,16]. These findings are in accordance with the theory of planned behaviour and the Precaution Adoption Process Model, which posits that individuals are not expected to begin the process of behaviour change until after they become aware that their current behaviour is putting their health at risk [19,20]. In this way, misperception of physical activity may be an important barrier to behaviour change, as it may result in individuals not recognising a need to increase their physical activity and being unaffected by physical activity promotion efforts.

To date, the majority of studies that have assessed awareness of physical activity have done so using scaled measures of self-rated physical activity that make no reference to physical activity guidelines, e.g., “In general, over the last year would you say you have been extremely active, moderately active, or not very active?” [14,15,17,21,22]. Responses have been interpreted dichotomously to suggest that individuals perceive themselves to be either “active” or “inactive”, and intermediate responses have been classified as “active” by default. Such methods are likely to introduce misclassification and the use of a direct measure of whether or not an individual perceives they are meeting the guidelines would be a substantial improvement [16]. Additionally, most studies have used self-reported measures of actual physical activity that have been shown to be prone to bias [14-16,23]. The use of an objective measure would provide a more valid operationalisation of awareness of physical activity [17,21,22].

In the present study, we assessed awareness of physical activity in a population-based sample of healthy middle-aged adults. We utilised a dichotomous measure of self-rated physical activity that makes reference to the physical activity guidelines, and we compared responses with a validated objective measure of physical activity. We also explored the associations between misperception of physical activity and various sociodemographic, biological, behavioural, and psychological factors. A deeper understanding of the factors associated with misperception could be important for determining why individuals choose to be active or inactive and might inform the development of effective strategies for promoting physical activity.

## Methods

### Design

This exploratory, cross-sectional study utilised data collected as part of a randomised controlled trial, the Feedback, Awareness and Behaviour (FAB) study [24]. All of the variables in the present study were collected at baseline, prior to randomisation and receipt of any intervention materials. The study obtained full ethical approval from the Cambridgeshire 2 Research Ethics Committee (reference number 07/Q0108/79). Written informed consent was obtained from each participant.

### Participants and setting

Participants of the FAB study were recruited from the Fenland Study, an ongoing population-based, observational study investigating the influence of lifestyle and genetic factors on the development of diabetes, obesity, and related metabolic disorders [25]. Patients born between 1950 and 1975 and registered with participating general practices in Cambridgeshire, UK were invited to take part. Exclusion criteria assessed by general practitioners included being diagnosed with diabetes, a terminal illness with a prognosis of less than one year, or a psychotic illness. Those who were pregnant or lactating, or unable to walk unaided were also excluded. Invitations to take part in the FAB study were sent to all participants who were scheduled to attend an assessment between September 2007 and August 2008. Those who developed a rash while wearing a combined heart rate monitor and accelerometer (explained in detail below) to measure free-living physical activity or who did not provide at least three days worth of complete physical activity data were excluded from the FAB study.

### Measures

During the Fenland Study, participants underwent a health assessment. Anthropometric (e.g., height and weight), clinical (e.g., blood pressure and pulse rate), and physical activity measurements (e.g., heart rate, movement, oxygen consumption at rest and during a sub-maximal treadmill test) were assessed by trained staff using standard operating procedures. An oral glucose tolerance test was administered, and two blood samples were taken to assess glucose levels and blood lipids. Demographics, medical history, and general lifestyle were assessed through self-report, and the validated SF-8™ Health Survey was completed [26]. At the end of the assessment, a single-piece monitor capable of measuring both acceleration and heart rate (Actiheart®) was used to objectively measure free-living physical activity for six days and nights continuously [27]. Physical activity level (PAL) was calculated as the ratio of total energy expenditure in a 24-hour period to basal metabolic rate. The average PAL over each day that a participant wore a monitor was calculated for participants who wore a monitor on three or more days and had at least 72 hours of complete data. The objectively measured average PAL was used in the assessment of awareness of physical activity (explained in detail below). Physical activity energy expenditure (PAEE) was calculated using branched equation modelling and both acceleration and heart rate data [28]. This approach has high validity for estimating the intensity of physical activity [29,30] and overcomes some of the key limitations associated with either accelerometers or heart rate monitors alone [27]. Self-reported physical activity was also measured during this time via the previously validated Recent Physical Activity Questionnaire [31].

Prior to the start of their Fenland Study health assessment, FAB study participants completed a baseline questionnaire, which included a measure of self-rated physical activity (explained in detail below). The measures were taken from the previously validated ProActive study questionnaires, which were largely based on the Theory of Planned Behavior and were amended where appropriate [19,32,33]. Time orientation (defined as the tendency to be motivated by either future or present goals in making decisions) was measured using a nine-item form of the validated Zimbardo Time Perspective Inventory [34]. Concern about physical activity was measured by asking participants, “How concerned are you about your level of physical activity?” Participants rated their concern on a 4-point scale, ranging from “not at all” to “very”. Worry about physical activity was measured by asking participants, “How often have you thought about your level of physical activity?” and “How often have thoughts about your physical activity level affected your mood?” Participants answered on a 4-point scale, ranging from “not at all” to “almost all of the time” [32,33].

Each of the following items included a statement that was evaluated on a 5-point response scale, ranging from “strongly disagree” to “strongly agree” [19,32,33]. Self-efficacy (e.g., “I am confident that I could be more physically active in the next two months, if I wanted to”) and response efficacy (e.g., “If I was more physically active in the next two months, it is likely that my health would improve”) were measured with two and four Likert items, respectively. Perceived importance (i.e., “Physical activity is important for maintaining good health”), subjective norm (i.e., “Most people who are important to me would want me to be more physically active”), perceived adequacy (i.e., I do enough physical activity to stay healthy), and intention to be more physically active (i.e., “I intend to be more physically active in the next two months”) each were measured using one Likert item.

**Figure 1:**
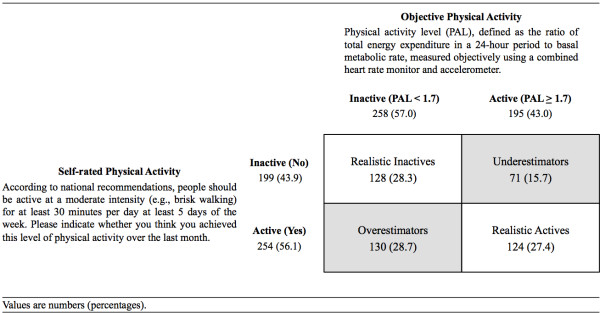
**Classification of participants into awareness groups (*n*** **= 453).**

In order to assess awareness of physical activity, we first informed participants that, according to national recommendations, people should be active at a moderate intensity (e.g., brisk walking) for at least 30 minutes per day at least 5 days of the week. Self-rated physical activity was then measured by asking participants to indicate whether they think they achieved this level of activity over the preceding month. We then classified participants’ objective physical activity as either inactive (PAL < 1.7) or active (PAL ≥ 1.7) in line with physical activity guidelines [2,3]. An habitual PAL ≥ 1.7 is associated with reduced risk of overweight, obesity, type 2 diabetes, and cardiovascular disease, and is approximately equivalent to 30 minutes of moderate to vigorous physical activity per day at least 5 days of the week [3]. Participants were grouped into a 2×2 table based upon the agreement between their self-rated and objective physical activity measures. This resulted in four awareness groups: overestimators, realistic inactives, underestimators, and realistic actives (see Figure 1).

### Analyses

All statistical analyses were conducted using STATA software [35] and two-tailed *p*-values with the predefined cut-off for statistical significance set at 0.05. Descriptive statistics (means, standard deviations, numbers, and percentages) were used to describe key demographic and health characteristics. Analyses were undertaken separately for inactive participants (overestimators and realistic inactives) and active participants (realistic actives and under-estimators) to establish correlates of misperception of physical activity. Univariable associations were assessed using Student’s *t*-tests for continuous variables and Pearson’s chi-square tests for categorical variables. Statistically significant variables were selected for inclusion in multivariable logistic regression models, adjusted for age and sex. Multicollinearity was checked and variance inflation factor statistics for each variable were less than 1.6. Variables that did not retain their significance were manually removed, one at a time, starting with the variable with the highest *p*-value, until a model containing only variables with significant associations was achieved. To distinguish associations between proximal and distal factors and to avoid overadjustment (i.e., the inclusion of variables hypothesised to be on the causal pathway), the psychological variables were modelled separately from the sociodemographic, biological, and behavioural variables.

## Results

Invitations to take part in the FAB study were mailed to 730 Fenland Study participants. Of the 544 (74.5%) individuals who were assessed for eligibility, 91 (16.7%) were missing baseline data on one or more of the independent variables of interest. Those with missing data were excluded from all analyses and did not differ from those included in the analyses according to age, sex, or body mass index (BMI; all *p* > 0.05).

### Sample characteristics

The mean (standard deviation) age of participants in the final sample (*n* = 453) was 47.0 (6.9) years, and 44.4% were male. Most obtained at least a secondary school level of education and were employed full-time (65.8%). On average, participants were overweight (65.1% had a BMI ≥ 25 kg/m^2^), with 25.4% classified as obese (a BMI >30 kg/m^2^). The majority were non-smokers (85.9%) who did not drink more than 10 units of alcohol per week (74.6%) and rated their overall health as either good, very good, or excellent (74.0%).

Figure 1 shows the classification of participants into four physical activity awareness groups. Approximately 55.7% of participants correctly assessed whether or not they were meeting the physical activity guidelines (28.3% realistic inactives and 27.4% realistic actives). Of the 258 (57.0%) participants who were objectively classified as inactive, 130 (50.4%) misperceived their physical activity by incorrectly stating that they were meeting the guidelines (overestimators). Of the 195 (43.0%) participants who were objectively classified as active, 71(36.4%) misperceived their physical activity by incorrectly stating that they were not meeting the guidelines (underestimators).

### Univariable associations

Table 1 shows the characteristics of participants by physical activity awareness groups. There were no differences between the groups according to age, sex, education, family history of diabetes, HbA_1c_ level, systolic blood pressure, smoking status, alcohol consumption, SF-8 physical summary, perceived importance, worry, and present time orientation. Among the inactive participants, overestimators were less likely than realistic inactives to be employed full-time. They also had a lower BMI and were more physically active than realistic inactives according to both objective and self-reported measures. Compared to realistic inactives, overestimators expressed significantly less intention to increase physical activity, subjective norm, response efficacy, self-efficacy, and concern. They also expressed greater perceived adequacy and are more oriented towards making goals for the future.

**Table 1:** **Participant characteristics categorized by misperception of physical activity within the inactive and active groups (*n*** **= 453)**

	**Inactive (*n*** **= 258)**			**Active (*n*** **= 195)**		
	**Overestimators**	**Realistic inactives**	***p*-value**	**Underestimators**	**Realistic actives**	***p*-value**
	**(*n*** **= 130)**	**(*n*** **= 128)**		**(*n*** **= 71)**	**(*n*** **= 124)**	
**Sociodemographic**						
Age (years)	47.7 (6.4)	47.3 (6.5)	0.68	46.2 (7.4)	46.3 (7.3)	0.95
Male, n (%)	20 (15.4)	25 (19.5)	0.38	58 (81.7)	98 (79.0)	0.66
Age ending full-time education (years)	18.0 (3.7)	18.2 (3.5)	0.66	18.0 (2.6)	18.0 (3.3)	0.97
Employed full-time, n (%)	59 (45.4)	79 (61.7)	<0.01	59 (83.1)	101 (81.5)	0.77
**Biological**						
Body mass index (kg/m^2^)	25.8 (4.9)	27.7 (5.5)	<0.01	29.5 (4.3)	27.1 (4.3)	<0.001
Family history of diabetes, n (%)	29 (22.3)	40 (31.3)	0.11	22 (31.0)	30 (24.2)	0.30
HbA_1c_ (mmol/mol)	35.0 (3.5)	35.3 (5.3)	0.63	37.2 (9.5)	35.9 (4.0)	0.26
VO_2_ max (ml/kg/min)	35.3 (7.9)	33.5 (7.8)	0.07	36.0 (6.7)	38.7 (6.7)	<0.01
Cholesterol ratio (mmol/L)	3.3 (0.9)	3.5 (1.0)	0.10	4.3 (1.4)	3.7 (1.1)	<0.001
Pulse rate (beats per minute)	66.8 (9.6)	68.6 (8.5)	0.12	71.7 (11.8)	66.1 (9.9)	<0.001
Systolic blood pressure (mmHg)	120.7 (14.9)	121.8 (16.2)	0.58	125.8 (12.4)	126.2 (13.6)	0.83
**Behavioural**						
Physical activity energy expenditure (kJ/kg/day)	40.0 (10.9)	35.4 (10.6)	<0.001	52.6 (13.4)	59.6 (16.4)	<0.01
Self-reported physical activity (MET h/day)	11.2 (6.6)	7.8 (5.4)	<0.001	9.4 (5.8)	15.8 (10.2)	<0.001
Current smoker, n (%)	15 (11.5)	14 (10.9)	0.88	16 (22.5)	19 (15.3)	0.21
Consume more than 10 units of alcohol per week, n (%)	28 (21.5)	19 (14.8)	0.54	21 (29.6)	47 (37.9)	0.22
**Psychological**						
Self-rated health good, very good, or excellent, n (%)	99 (76.2)	90 (70.3)	0.29	42 (59.2)	104 (83.9)	<0.001
SF-8 physical summary	52.6 (7.3)	51.2 (7.4)	0.15	52.1 (6.3)	53.2 (6.5)	0.26
SF-8 mental summary	50.0 (8.2)	48.1 (8.0)	0.06	48.1 (9.1)	50.9 (8.1)	<0.05
Intention (1 to 5)	3.2 (0.8)	3.6 (0.8)	<0.001	3.5 (1.0)	3.2 (0.9)	<0.05
Perceived adequacy (1 to 5)	3.6 (0.9)	2.8 (0.9)	<0.001	2.5 (0.8)	3.8 (1.0)	<0.001
Subjective norm (1 to 5)	2.8 (0.9)	3.3 (0.9)	<0.001	3.6 (0.9)	2.8 (1.0)	<0.001
Perceived importance (1 to 5)	4.5 (0.5)	4.5 (0.5)	0.48	4.4 (0.5)	4.6 (0.6)	0.07
Response efficacy (1 to 5)	3.7 (0.7)	4.0 (0.5)	<0.001	4.1 (0.5)	3.7 (0.8)	<0.01
Self-efficacy (1 to 5)	3.6 (0.8)	3.8 (0.7)	<0.05	3.8 (0.7)	3.7 (0.7)	0.46
Worry (1 to 4)	2.8 (0.8)	2.9 (0.9)	0.38	2.7 (0.8)	2.8 (1.0)	0.36
Concern (1 to 4)	2.2 (1.0)	2.6 (0.8)	<0.001	2.7 (0.8)	2.2 (1.0)	<0.001
Present orientation (1 to 5)	2.8 (0.8)	2.8 (0.7)	0.86	2.9 (0.8)	2.7 (0.8)	0.30
Future orientation (1 to 5)	3.6 (0.7)	3.3 (0.7)	0.004	3.4 (0.7)	3.4 (0.7)	0.65

Values are means (standard deviations) unless otherwise specified. HbA_1c_: Glycated haemoglobin. VO_2_ max: maximal oxygen consumption. Cholesterol ratio: total cholesterol divided by high-density lipoprotein cholesterol.

Among the active participants, underestimators had a higher BMI than realistic actives. They also had a lower VO_2_ max, a higher cholesterol ratio and pulse rate, and were less physically active than realistic actives according to both objective and self-reported measures. Compared to realistic actives, underestimators expressed significantly greater intention to increase physical activity, subjective norm, response efficacy, and concern. They also expressed less perceived adequacy, scored lower on the SF-8 mental summary scale, and were less likely to rate their overall health as good, very good, or excellent.

### Multivariable associations

Table 2 shows the results of logistic regression models of misperception of physical activity on the sociodemographic, biological, and behavioural variables (Model 1) and on the psychological variables (Model 2), each adjusted for age and sex. Among the inactive participants, the results of Model 1 show that the multivariable associations with overestimation were similar to the univariable associations. Model 2 shows that those with less intention to increase physical activity and response efficacy, and those with greater perceived adequacy and future-orientation, were more likely to overestimate their physical activity.

**Table 2:** Multivariable logistic regression models showing odds of misperceiving physical activity, adjusted for age and sex, within the inactive and active groups

	**Inactive (*n*** **= 258)**			**Active (*n*** **= 195)**		
	**Overestimators (*n*** **= 130) vs. Realistic inactives (*n*** = 128)*			**Underestimators (*n*** **= 71) vs. Realistic actives (*n*** = 124)*		
	**Odds ratio**	**95% CI**	***p*-value**	**Odds ratio**	**95% CI**	***p*-value**
**Sociodemographic, biological, and behavioural (Model 1)**						
Employed full-time, n (%)	0.39	0.22 to 0.69	<0.01	**–**	**–**	**–**
Body mass index (kg/m^2^)	0.95	0.90 to 1.00	<0.05	1.09	1.01 to 1.19	<0.05
VO_2_ max (ml/kg/min)	**–**	**–**	**–**	Removed from model		
Cholesterol ratio (mmol/L)	**–**	**–**	**–**	Removed from model		
Pulse rate (beats per minute)	**–**	**–**	**–**	1.05	1.02 to 1.09	<0.01
Physical activity energy expenditure (kJ/kg/day)	1.03	1.00 to 1.06	<0.05	Removed from model		
Self-reported physical activity (MET h/day)	1.13	1.07 to 1.19	<0.001	0.88	0.82 to 0.93	<0.001
**Psychological (Model 2)**						
Self-rated health good, very good, or excellent, n (%)	**–**	**–**	**–**	Removed from model		
SF-8 mental summary	**–**	**–**	**–**	Removed from model		
Intention (1 to 5)	0.69	0.48 to 0.99	<0.05	Removed from model		
Perceived adequacy (1 to 5)	2.23	1.63 to 3.04	<0.001	0.28	0.18 to 0.43	<0.001
Subjective norm (1 to 5)	Removed from model			1.53	1.00 to 2.35	<0.05
Response efficacy (1 to 5)	0.53	0.31 to 0.91	<0.05	Removed from model		
Self-efficacy (1 to 5)	Removed from model			**–**	**–**	**–**
Concern (1 to 4)	Removed from model			Removed from model		
Future orientation (1to 5)	1.54	1.03 to 2.31	<0.05	**–**	**–**	**–**

*Reference group. VO_2_ max: maximal oxygen consumption. Cholesterol ratio: total cholesterol divided by high-density lipoprotein cholesterol. CI: confidence interval.

Among the active participants, the results of model 1 show that a high BMI and pulse rate, and a low self-reported physical activity were associated with a greater likelihood of underestimation. Model 2 shows that those with less perceived adequacy and greater subjective norm were more likely to underestimate their physical activity.

## Discussion

This is the first study to assess awareness of physical activity using a dichotomous measure of self-rated physical activity that specifically asked about adherence to physical activity guidelines and compared responses with a precise objective measure. We found that among the 57.0% of adults who were inactive, 50.4% incorrectly perceived that they were achieving the recommended level of physical activity. Our findings are similar to those of previous studies, which show that the prevalence of overestimation is between 46% and 61% [14-17]. Additionally, the proportion of those who were active, but who underestimate their physical activity was low (36.4%). Considering that this small body of research has been limited by a reliance on potentially biased assessments of awareness of physical activity, our confirmation of previous findings is noteworthy and indicates that physical activity misperception is a common phenomenon across multiple populations.

Compared to those who correctly perceived themselves to be inactive, overestimators had a lower BMI and engaged in more physical activity according to both self-reported and objective measures, although not enough to be classified as active. They were also less likely to be employed full-time. In contrast, individuals who underestimated their physical activity had a higher BMI and pulse rate, and engaged in less physical activity than individuals who correctly perceived themselves to be active. These findings are in line with previous research that suggests overestimators have more positive indicators of health compared to underestimators [15-17]. One plausible explanation for these results is that overestimators have an optimistic bias and they assume that they are sufficiently active simply because they have more favourable health characteristics [17]. For example, the link between physical activity and weight is well known. Overestimators may therefore take their lower BMI as a signal that their level of physical activity is adequate, regardless of whether or not it actually is. For underestimators, this mechanism may operate in reverse, with less favourable health characteristics signalling that they are insufficiently active. In order to further explore these associations, and to establish the temporality of the proposed mechanism, longitudinal studies that assess changes in awareness of physical activity and health characteristics are needed.

It is important to highlight that although overestimators appear to be healthier than those who are realistic about their inactivity, on average they were overweight and their physical activity level was inadequate. This places them at increased health risk, which may be worsened by their inaccurate belief that they do enough physical activity to stay healthy [1]. Overestimators are also less inclined to believe that physical activity has beneficial effects, and they express less intention to increase their physical activity than those who are realistic about their inactivity. Interestingly, they are also more motivated by future goals than realistic inactives. Compared to those who correctly perceived themselves to be active, underestimators had less of a belief that they do enough physical activity to stay healthy, but they did have a strong belief that those who are important want them to be more physically active. These results replicate previously shown associations between overestimation and intentions [14,16], as well as between underestimation and subjective norm [24], suggesting that they are unlikely to be due to chance. Within the framework of health behaviour theory, each of these findings suggest that overestimators are less likely to increase their physical activity than realistic inactives, and that underestimators are more likely to increase their activity than realistic actives [19,20]. Additional research is necessary to determine the association between misperception of physical activity and behaviour, and between changes in awareness of physical activity and changes in behaviour.

Many theory-based physical activity interventions target the psychological factors that are the hypothesised antecedents of behaviour change [9,36]. Our results suggest that this may be ineffective in the presence of misperception of physical activity, which could act as a barrier to behaviour change. The provision of personalised feedback about physical activity following objective measurement might facilitate an accurate perception of physical activity [24,37,38]. In turn, this might stimulate intention to increase physical activity, which ultimately may lead to positive changes in behaviour. This pathway is consistent with several health behaviour theories [19,20] and may be part of the mechanism underlying the effectiveness of pedometers [39,40]. Currently, there are efforts to utilise the rapidly expanding world of inexpensive measurement devices, smart phones, and tablet computers to objectively measure the physical activity of large numbers of individuals and provide them with instantaneous, personalised feedback [41]. However, studies of the effects of personalised feedback on awareness of physical activity, intention to increase physical activity, and behaviour have been limited by small sample sizes and imprecise outcome measurement, and large randomised controlled trials with objective outcome assessment would advance research in this area [42-45].

Strengths of this study include the large population-based sample and the use of a dichotomous measure of self-rated physical activity, a validated and objective measure of actual physical activity, and a range of well-assessed correlates. However, the results of this study should be considered within its limitations. Although our measure of awareness of physical activity represents an improvement in the assessment of the construct, the time frames of the self-rated (the past month) and objective (the subsequent six days and nights) physical activity measures did not overlap. The intent of both measures was to capture habitual behaviour, but measures that have matching or overlapping time frames may be an improvement. It should also be acknowledged that random error or social desirability could have influenced the measurement of self-rated and objective physical activity and may have influenced our assessment of awareness of physical activity. Additionally, we relied on single-item measures of self-rated physical activity, perceived importance, subjective norm, perceived adequacy, and intention to be physically active. A more comprehensive assessment of the psychological antecedents of behaviour change is necessary to fully understand what role psychological variables might play in facilitating awareness of physical activity. Participants were from one location in the United Kingdom and were physically and psychologically healthy. Therefore, the results might not generalise to other settings or to those who are less healthy. Finally, the cross-sectional study design prohibits the establishment of any temporal associations.

## Conclusions

In this population-based sample of middle-aged adults, half of those who were inactive misperceived their physical activity by overestimating the amount of activity they engaged in. Overestimation was associated with a lower BMI, higher levels of physical activity according to objective and self-reported measures, lower intention to increase physical activity and response efficacy. These results are in accordance with previous research [14-17]. This is significant considering that the present study is the first to assess awareness of physical activity using a dichotomous measure of self-rated physical activity that asked individuals if they were achieving the recommended level of physical activity and compared responses with a precise objective measure. Our results imply that public health messages aimed at promoting physical activity among the general population are unlikely to reach inactive adults who believe their level of physical activity is adequate. Increasing awareness of physical activity and adapting public health messages to underscore that relatively healthy, normal weight and overweight individuals who are moderately active can still garner health benefits from increasing their physical activity to the recommend level may be important in promoting physical activity. This study emphasises the need for more research into the association between misperception of physical activity and behaviour, as well as the effects of feedback on awareness of physical activity, intention to increase physical activity, and behaviour.

## Abbreviations

OR: Odds ratio; CI: Confidence interval; FAB: The feedback, awareness and behaviour study; PAL: Physical activity level; PAEE: Physical activity energy expenditure; BMI: Body mass index.

## Competing interests

The authors declare that they have no competing interests.

## Authors’ contributions

JGG, CW, KC, SJG, and EMFvS defined the research question. JGG wrote the statistical analysis plan, conducted the statistical analyses, and drafted the manuscript. JGG, KC, SS, SJG, and EMFvS have contributed to the interpretation of the results and were involved in critical revisions. CW, SS, SJG, and EMFvS contributed to the development of the FAB study and the measures utilised. CW created the study materials and coordinated the study throughout. All authors read and approved the final manuscript.

## Pre-publication history

The pre-publication history for this paper can be accessed here:

http://www.biomedcentral.com/1471-2458/14/421/prepub

## References

[B1] Lee I-M Shiroma EJ Lobelo F Puska P Blair SN Katzmarzyk PT Effect of physical inactivity on major non-communicable diseases worldwide: An analysis of burden of disease and life expectancy Lancet 2012 380 219 29 10.1016/S0140-6736(12)61031-9 22818936 PMC3645500

[B2] Department of Health, Physical Activity, Health Improvement and Prevention At Least Five a Week: Evidence on the Impact of Physical Activity and Its Relationship to Health 2004 London: A Report from the Chief Medical Officer

[B3] FAO/WHO/UNU Human Energy Requirements: Report of a Joint FAO/WHO/UNU Expert Consultation 2001 Rome: FAO Food and Nutrition Technical Report Series

[B4] Bull FC The Expert Working Groups Physical Activity Guidelines in the UK: Review and Recommendations 2010 Loughborough: School of Sport, Exercise and Health Sciences, Loughborough University

[B5] Change4Life: Move More http://www.nhs.uk/Change4Life/

[B6] NHS Choices: Live Well http://www.nhs.uk/LiveWell/

[B7] Craig R Mindell J Hirani V Health Survey for England 2008, Volume 1: Physical Activity and Fitness 2009 London: The Health and Social Care Information Centre 395

[B8] Tucker JM Welk GJ Beyler NK Physical activity in U.S. Adults: Compliance with the Physical Activity Guidelines for Americans Am J Prev Med 2011 40 454 61 10.1016/j.amepre.2010.12.016 21406280

[B9] Van Sluijs EMF van Poppel MNM van Mechelen W Stage-based lifestyle interventions in primary care: Are they effective? Am J Prev Med 2004 26 330 43 15110061 10.1016/j.amepre.2003.12.010

[B10] Hillsdon M Foster C Thorogood M Interventions for promoting physical activity Cochrane Database Syst Rev 2005 1 CD003180 15674903 10.1002/14651858.CD003180.pub2 PMC4164373

[B11] Müller-Riemenschneider F Reinhold T Nocon M Willich SN Long-term effectiveness of interventions promoting physical activity: A systematic review Prev Med (Baltim) 2008 47 354 68 10.1016/j.ypmed.2008.07.006 18675845

[B12] Orrow G Kinmonth A-L Sanderson S Sutton S Effectiveness of physical activity promotion based in primary care: Systematic review and meta-analysis of randomised controlled trials BMJ 2012 344 e1389 10.1136/bmj.e1389 22451477 PMC3312793

[B13] Bauman AE Reis RS Sallis JF Wells JC Loos RJF Martin BW Correlates of physical activity: Why are some people physically active and others not? Lancet 2012 380 258 71 10.1016/S0140-6736(12)60735-1 22818938

[B14] Ronda G Van Assema P Brug J Stages of change, psychological factors and awareness of physical activity levels in The Netherlands Health Promot Int 2001 16 305 14 10.1093/heapro/16.4.305 11733449

[B15] Lechner L Bolman C Van Dijke M Factors related to misperception of physical activity in The Netherlands and implications for health promotion programmes Health Promot Int 2006 21 104 12 10.1093/heapro/dal011 16641132

[B16] Van Sluijs EM Griffin SJ Van Poppel MNM A cross-sectional study of awareness of physical activity: Associations with personal, behavioral and psychosocial factors Int J Behav Nutr Phys Act 2007 9 1 9 10.1186/1479-5868-4-53 PMC2186356 17996060

[B17] Watkinson C van Sluijs EM Sutton S Hardeman W Corder K Griffin SJ Overestimation of physical activity level is associated with lower BMI: A cross-sectional analysis Int J Behav Nutr Phys Act 2010 7 68 10.1186/1479-5868-7-68 20854659 PMC2954949

[B18] Thompson D Batterham AM Markovitch D Dixon NC Lund AJS Walhin J-P Confusion and conflict in assessing the physical activity status of middle-aged men PLoS One 2009 4 e4337 10.1371/journal.pone.0004337 19183812 PMC2629570

[B19] Ajzen I The theory of planned behavior Organ Behav Hum Decis Process 1991 50 179 211 10.1016/0749-5978(91)90020-T

[B20] Weinstein ND The precaution adoption process Heal Psychol 1988 7 355 386 10.1037//0278-6133.7.4.355 3049068

[B21] Corder K van Sluijs EM McMinn AM Ekelund U Cassidy A Griffin SJ Perception versus reality: Awareness of physical activity levels of British children Am J Prev Med 2010 38 1 8 10.1016/j.amepre.2009.08.025 20117551 PMC3746297

[B22] Corder K van Sluijs EM Goodyer I Ridgway CL Steele RM Bamber D Dunn V Griffin SJ Ekelund U Physical activity awareness of British adolescents Arch Pediatr Adolesc Med 2011 165 603 9 10.1001/archpediatrics.2011.94 24187480 PMC3812705

[B23] Helmerhorst HJF Brage S Warren J Besson H Ekelund U A systematic review of reliability and objective criterion-related validity of physical activity questionnaires Int J Behav Nutr Phys Act 2012 9 103 10.1186/1479-5868-9-103 22938557 PMC3492158

[B24] Watkinson C van Sluijs EMF Sutton S Marteau T Griffin SJ Randomised controlled trial of the effects of physical activity feedback on awareness and behaviour in UK adults: The FAB study protocol [ISRCTN92551397] BMC Public Health 2010 10 144 10.1186/1471-2458-10-144 20298560 PMC2859395

[B25] Rolfe E Loos R Druet C Stolk R Ekelund U Griffin S Forouhi N Wareham N Ong K Association between birth weight and visceral fat in adults Am J Clin Nutr 2010 92 347 352 10.3945/ajcn.2010.29247 20519560

[B26] Ware J Kosinski M Dewey J Gandek B How to Score and Interpret Single-Item Health Status Measures: A Manual for Users of the SF-8 Health Survey 2001 Lincoln, RI: QualityMetric Incorporated

[B27] Brage S Brage N Franks PW Ekelund U Wareham NJ Reliability and validity of the combined heart rate and movement sensor Actiheart Eur J Clin Nutr 2005 59 561 70 10.1038/sj.ejcn.1602118 15714212

[B28] Brage S Brage N Franks PW Ekelund U Wong M-Y Andersen LB Froberg K Wareham NJ Branched equation modeling of simultaneous accelerometry and heart rate monitoring improves estimate of directly measured physical activity energy expenditure J Appl Physiol 2004 96 343 51 12972441 10.1152/japplphysiol.00703.2003

[B29] Strath SJ Brage S Ekelund U Integration of physiological and accelerometer data to improve physical activity assessment Med Sci Sport Exerc 2005 37 S563 S571 10.1249/01.mss.0000185650.68232.3f 16294119

[B30] Thompson D Batterham A Bock S Robson C Stokes K Assessment of low-to-moderate intensity physical activity thermogenesis in young adults using synchronized heart rate and accelerometry with branched-equation modeling J Nutr 2006 136 1037 1042 16549471 10.1093/jn/136.4.1037

[B31] Besson H Brage S Jakes R Ekelund U Wareham N Estimating physical activity energy expenditure, sedentary time, and physical activity intensity by self-report in adults Am J Clin Nutr 2010 91 106 114 10.3945/ajcn.2009.28432 19889820

[B32] Williams K Prevost AT Griffin S Hardeman W Hollingworth W Spiegelhalter D Sutton S Ekelund U Wareham N Kinmonth AL The ProActive trial protocol - a randomised controlled trial of the efficacy of a family-based, domiciliary intervention programme to increase physical activity among individuals at high risk of diabetes [ISRCTN61323766] BMC Public Health 2004 4 48 10.1186/1471-2458-4-48 15491494 PMC526256

[B33] Sutton S French DP Hennings SJ Mitchell J Wareham NJ Griffin S Hardeman W Kinmonth AL Eliciting salient beliefs in research on the theory of planned behaviour: The effect of question wording Curr Psychol 2003 22 234 251 10.1007/s12144-003-1019-1

[B34] Crockett RA Weinman J Hankins M Marteau T Time orientation and health-related behaviour: measurement in general population samples Psychol Health 2009 24 333 350 10.1080/08870440701813030 20204997 PMC2657323

[B35] StataCorp Stata Statistical Software: Release 12 2011

[B36] Hardeman W Sutton S Griffin S Johnston M White A Wareham NJ Kinmonth AL A causal modelling approach to the development of theory-based behaviour change programmes for trial evaluation Health Educ Res 2005 20 676 87 10.1093/her/cyh022 15781446

[B37] DiClemente CC Marinilli AS Singh M Bellino LE The role of feedback in the process of health behavior change Am J Health Behav 2001 25 217 27 10.5993/AJHB.25.3.8 11322620

[B38] Proper KI van der Beek AJ Hildebrandt VH Twisk JWR van Mechelen W Short term effect of feedback on fitness and health measurements on self reported appraisal of the stage of change Br J Sports Med 2003 37 529 34 10.1136/bjsm.37.6.529 14665593 PMC1724704

[B39] Bravata D Smith-Spangler C Sundaram V Gienger A Lin N Lewis R Stave C Olkin I Sirard J Using pedometers to increase physical activity and improve health: A systematic review JAMA 2007 298 2296 2304 10.1001/jama.298.19.2296 18029834

[B40] Tudor-Locke C Lutes L Why do pedometers work?: a reflection upon the factors related to successfully increasing physical activity Sport Med 2009 39 981 93 10.2165/11319600-000000000-00000 19902981

[B41] King AC Ahn DK Oliveira BM Atienza AA Castro CM Gardner CD Promoting physical activity through hand-held computer technology Am J Prev Med 2008 34 138 42 10.1016/j.amepre.2007.09.025 18201644 PMC2715220

[B42] Marcus B Bock B Pinto B Efficacy of an individualised, motivationally-tailored physical activity intervention Ann Behav Med 1998 20 174 180 10.1007/BF02884958 9989324

[B43] Kroeze W Werkman A Brug J A systematic review of randomized trials on the effectiveness of computer-tailored education on physical activity and dietary behaviors Ann Behav Med 2006 31 205 23 10.1207/s15324796abm3103_2 16700634

[B44] Smeets T Brug J de Vries H Effects of tailoring health messages on physical activity Health Educ Res 2008 23 402 13 10.1093/her/cyl101 17032705

[B45] Van Stralen MM de Vries H Mudde AN Bolman C Lechner L The long-term efficacy of two computer-tailored physical activity interventions for older adults: main effects and mediators Heal Psychol 2011 30 442 52 10.1037/a0023579 21639638

